# Subjective Global Assessment and Handgrip Strength as Predictive Factors in Patients with Liver Cirrhosis

**DOI:** 10.1155/2017/8348390

**Published:** 2017-07-18

**Authors:** Maria Ciocîrlan, Andreea Ruxandra Cazan, Mihaela Barbu, Mircea Mănuc, Mircea Diculescu, Mihai Ciocîrlan

**Affiliations:** ^1^Gastroenterology Department, Fundeni Clinical Institute, Bucharest, Romania; ^2^“Carol Davila” University of Medicine and Pharmacy, Bucharest, Romania; ^3^“Victor Babes” Hospital, Bucharest, Romania; ^4^“Agrippa Ionescu” Clinical Emergency Hospital, Bucharest, Romania

## Abstract

**Background and Aims:**

Malnutrition is common in patients with chronic liver disease. We aimed to evaluate malnutrition assessment tools in predicting severity and survival of patients with liver cirrhosis.

**Material and Methods:**

We examined patients with liver cirrhosis. Nutritional evaluation was performed on admission, using subjective global assessment (SGA), handgrip strength (HGS), and anthropometry. Patients were followed up for 6 months.

**Results:**

We included 100 patients, 72 men, with mean age of 59.2 years. According to disease severity, patients were 23% Child-Pugh A, 46% Child-Pugh B, and 31% Child-Pugh C. SGA and HGS significantly correlated with Child-Pugh, MELD, and MELD-Na scores on admission. At 6 months follow-up, 80.4% (78 of 97) of patients survived, while 3 patients were lost from observation. Survival was predicted by SGA (1 death in 32 patients SGA A, 8 deaths in 46 patients SGA B, and 9 deaths in 19 patients SGA C, *p* = 0.001) and HGS (25.1 ± 8.5 in deceased versus 30.6 ± 10.9 in survivors, *p* = 0.046). The mean BMI and MAMC values did not significantly differ between patients who survived or were deceased at 6 months.

**Conclusion:**

HGS and SGA may predict severity and short-term survival in cirrhotic patients.

## 1. Introduction

Worldwide, malnutrition is common in patients with liver cirrhosis and has a negative impact on both morbidity and mortality [1]. Depending on the method of evaluation, the prevalence is thought to be of about 20% in patients with compensated disease, whereas in decompensated liver cirrhosis, it can reach 60% or more [2].

In advanced liver disease, the etiology of malnutrition may have many causes: poor energetic intake due to anorexia, a proinflammatory state, and the presence of ascites [3, 4]. The latter impedes an adequate oral intake and increases abdominal pressure contributing to early satiety, sensation of fullness, or even vomiting. Often, cirrhotic patients have metabolic disturbances (e.g., low zinc or magnesium levels) that can promote taste alteration [4]. Once hepatic encephalopathy is installed, a low-protein diet is still recommended by some physicians, sometimes with severe protein restriction [3, 4]. All of the above lead to loss of fat mass but, most importantly, to skeletal muscle waste (sarcopenia) [3, 4].

Body mass index (BMI) and subjective global assessment (SGA) are both used for evaluation of malnutrition. Nevertheless, SGA may underestimate malnutrition in decompensated cirrhosis, has poor interobserver agreement, and requires patient's cooperation (impaired in overt hepatic encephalopathy) [3, 5, 6]. BMI underestimates malnutrition in patients with ascites or peripheral edema.

Sarcopenia is closely correlated to development of malnutrition in cirrhosis [4] and may be evaluated by either muscle mass measurements or muscle strength or both.

Muscle mass estimation can be done using the mid-arm muscle circumference (MAMC), bioelectrical impedance analysis (BIA), dual energy X-ray absorptiometry (DEXA), or by CT scan.

MAMC is calculated based on the anthropometric measurement of the triceps skinfold (TSF) and mid-arm circumference (MAC) on the nondominant arm. MAMC has been deemed inaccurate in patients with generalized edema [7, 8].

BIA is based on the fact that electrical current is conducted faster in water and fat-free tissues and slower in fat tissue [9]. Based on the measured electrical current transmitted through tissues, one can estimate the proportion of fat-free mass and fat mass. Results are influenced by physical activity, hydration status, diuretic use, fluid retention, and eating or drinking before the examination. BIA can be inaccurate in cirrhotic patients with generalized edema [8].

DEXA is considered extremely accurate in evaluating nutritional status in cirrhosis. Depending on the body composition (bone, fat, and lean mass), the energy photons pass through the body in variable amounts. This characteristic can be used to identify tissue composition [10]. DEXA has a great reproducibility, but it is expensive and unavailable in daily clinical practice [8].

Measurement of the psoas muscle area on CT scan images has proven reliable in evaluating sarcopenia in cirrhotic patients [11, 12]. The skeletal muscle index measured at the level of the third lumbar vertebrae has not yet entered clinical practice as CT scans may not be repeated as often as one would need to assess nutritional status in a patient with cirrhosis.

Besides muscle mass, muscle strength has been proposed as a useful predictor of sarcopenia. As ammonia is converted to glutamine in the skeletal muscles, the handgrip strength (HGS) may be of particular interest in patients with hepatic encephalopathy [13]. Muscle strength is estimated by measuring the HGS of the arm by using a dynamometer [14, 15].

We aimed to assess the value of current clinical malnutrition assessment tools (SGA, BMI, MAMC, and HGS) in predicting severity and survival of patients with liver cirrhosis.

## 2. Methods

We prospectively evaluated all patients with liver cirrhosis admitted in Gastroenterology and Hepatology Clinic, Fundeni Clinical Institute, between March 2015 and March 2016.

Admission motives included occurrence of complications (ascites, spontaneous bacterial peritonitis, hepatorenal syndrome, hepatic encephalopathy, and gastrointestinal bleeding) or follow-up. Patients with suspected or confirmed hepatocarcinoma were excluded.

We evaluated the severity of liver cirrhosis using the Child-Pugh and MELD-Na scores at admission in all patients [16].

Nutritional assessment was performed in all patients using SGA score on the day of admission [17]. Based on history and physical examination of the patient, patients were subjectively rated as well nourished (A), moderately malnourished (B), or severely malnourished (C). The examiner asked every patient about his/her involuntary weight loss and change in dietary intake in the past 6 months and 2 weeks, the presence of gastrointestinal symptoms for more than 2 weeks (nausea, vomiting, diarrhea, and anorexia), and his/her performance status. Physical examination assessed on a 4-point scale (o to 3) the severity of subcutaneous fat loss, muscle wasting, ankle, and sacral edema as well as ascites.

Anthropometrical measurements were performed in all patients: BMI, TSF, and MAC. The mid-arm muscle circumference was calculated using the formula MAMC = MAC − 3.14∗(TSF) [18].

We measured the HGS of the dominant hand in all patients, using a Jamar dynamometer. The patient was asked to grasp the dynamometer handles with his/her hand and squeeze them with maximum strength, repeating three times. This translated to a proportional indicator movement on a circular scale which retained the maximum strength value (in kilogram force) with a peak hold needle.

Patient medical records were reviewed at 6 months to check for complications, death, or liver transplantation. If there were no new data at 6 months, patients were contacted by telephone.

Quantitative variables were presented as mean ± standard deviation (SD). Categorical variables were presented as absolute values and percentages. Comparison between groups were done using chi-square and Kruskal-Wallis test, while correlation between quantitative variables were done using Pearson *r* test. SPSS 16.0 was used for statistical analysis. A 2-tailed *p* value less than 0.05 was considered statistically significant.

The study was approved by the Hospital Ethics Committee.

## 3. Results

We included 100 patients. Their demographic characteristics as well as complications, severity scores, and survival data are presented in Table 1.

The mean HGS value for men was significantly higher than that for women (34.1 ± 10.6 in men versus 19.8 ± 4.9 in women, *p* < 10^−3^).

**Figure 1:**
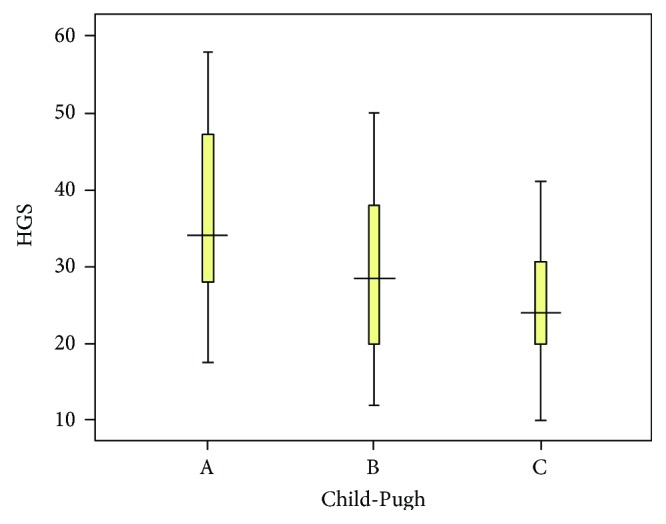
HGS mean and confidence interval distribution among Child-Pugh classes.

Comparison between distribution of SGA scores and the mean values of BMI, MAMC, and HGS among Child-Pugh groups are shown in Table 2. HGS mean values with corresponding confidence intervals are illustrated in Figure 1.

Comparative distribution of mean MELD and MELD-Na scores among the three SGA classes are shown in Table 3. Correlations between MELD, MELD-Na and BMI, MAMC, and HGS are shown in Table 4.

SGA score and HGS mean values were significantly higher and lower, respectively, with increasing cirrhosis severity as estimated by Child-Pugh class, MELD, and MELD-Na scores.

Survival at 6 months was 80.4% (78 of 97 patients). 3 patients were lost to follow-up. 5 patients had undergone successful liver transplantation.

Survival at 6 months was predicted by Child-Pugh class (no deaths in 23 Child-Pugh A patients, 8 of 46 Child-Pugh B patients, and 11 of 28 Child-Pugh C patients, *p* = 0.002), MELD score (16.5 ± 7.2 in deceased versus 12.7 ± 5.4 in survivors, *p* = 0.017), and MELD-Na score (18.7 ± 7.8 in deceased versus 14.4 ± 5.9 in survivors, *p* = 0.029).

The 6-months survival was also predicted by SGA (1 death in 32 patients SGA A, 8 deaths in 46 patients SGA B, and 9 deaths in 19 patients SGA C, *p* = 0.001) and HGS (25.1 ± 8.5 in deceased versus 30.6 ± 10.9 in survivors, *p* = 0.046).

The mean BMI and MAMC values did not significantly differ between patients who deceased or survived at 6 months (BMI 26.3 ± 6.4 in deceased versus 27.1 ± 4.9 in survivors, *p* = 0.406 and MAMC 24.8 ± 3.8 in deceased versus 26.3 ± 4.6 in survivors, *p* = 0.217).

## 4. Discussions

We included hospitalized patients with liver cirrhosis. Most patients were men (72%), had alcohol abuse as etiological factor (61%), and had decompensated disease (Child-Pugh B or C, 77%).

Apart from SGA where allocation in categories is self-explanatory as to the presence of malnutrition, we did not use BMI, MAMC, and HGS as tools to evaluate the nutritional status but rather as predictive factors for cirrhosis severity and prognosis.

Mean BMI values did not predict cirrhosis severity (Child-Pugh class, MELD, and MELD-Na) or 6 months survival. The mean BMI values were identical in Child-Pugh A and C classes, most probably in relation to fluid overload with ascites and edema in decompensated cirrhosis. This confirms what has been described by other authors [8, 15].

One study found a positive correlation between TSF and MAC and the severity of the liver disease [19]. 4% of their patients were Child-Pugh C and 59% had ascites. We used MAMC (which is derived from MAC and TSF) and did not find any correlation between MAMC and the severity of liver disease. One explanation may be that 31% of our patients were Child-Pugh C and 75% had ascites. Fluid overload with ascites and peripheral edema was more frequent in our study group which can explain overestimation of nutritional status by MAMC and lack of correlation with disease severity.

66% of our patients were malnourished (SGA B or C), higher than the reported 28% percentage in another similar study [14]. The difference may be partly explained by the fact that we included patients with more severe disease (our study—23% Child-Pugh A, 46% Child-Pugh B, and 31% Child-Pugh C and their study—88% Child-Pugh A, 12% Child-Pugh B, and no patients Child-Pugh C). Nevertheless, our patients were twice as malnourished when compared with the abovementioned study, as 8 of 23 (34.8%) Child-Pugh A patients and 37 of 69 (53.6%) Child-Pugh A and B patients were SGA B or C.

In our study population, SGA significantly correlated with cirrhosis prognostic scores (Child-Pugh, MELD, and MELD-Na) and predicted 6 months survival. Two studies on more than 150 patients with liver cirrhosis also found a significant correlation between SGA and Child-Pugh score (*p* < 0.05) [19, 20].

Similarly, HGS significantly correlated with cirrhosis prognostic scores (Child-Pugh, MELD, and MELD-Na) and could predict 6 months survival.

In a recent study by Gaikwad et al. [21], 80 patients with alcoholic liver disease with a mean MELD score of 10.50 ± 2.67 were followed for 3 months [21]. 11 patients died during the follow-up. A significant correlation was noted between HGS and Child-Pugh score (*r* = −0.606, *p* ≤ 0.0012) and MELD score (*r* = −0.394, *p* ≤ 0.001). Mean HGS was significantly lower in patients who died during follow-up (18.04 ± 4.82 in deceased versus 24.23 ± 5.86 in survivors, *p* = 0.001). We have shown similar results, with significantly lower HGS in patients who died during follow-up (25.1 ± 8.5 in deceased versus 30.6 ± 10.9 in survivors, *p* = 0.046).

In contrary to these results, a study by Fernandes et al. [15] showed no correlation between HGS and Child-Pugh classification. They included patients with HCV (43.4%) and alcoholic etiology (25.6%) and mostly patients with compensated liver cirrhosis (91 of 129 patients were Child-Pugh A, 27 were Child-Pugh B, and only 9 were Child-Pugh C). 54% were men. They tested the nondominant hand and showed that HGS did not significantly decrease with increasing severity of liver cirrhosis (24.7 ± 11.2 in Child-Pugh A, 26.6 ± 14.3 in Child-Pugh B, and 21.3 ± 11.7 in Child-Pugh C, *p* = 0.510).

The discrepancy between the results of these studies (ours and Gaikwad et al. [21] versus Fernandes et al. [15]) may come from the fact that we tested HGS of the dominant hand, similar to Gaikwad et al. [21]. This might also explain why we obtained higher mean HGS values than what other studies have reported (35.6 ± 12.4 for Child-Pugh A, 29.3 ± 10.1 for Child-Pugh B, and 25.3 ± 7.8 for Child-Pugh C, *p* = 0.007). However, it has been shown that there are no significant differences between the HGS values of the dominant and nondominant hands in normal subjects [22].

Moreover, 72% of our patients were males, with significantly higher HGS (34.1 ± 10.6 in men versus 19.8 ± 4.9 in women, *p* < 10^−3^). However, in our paper, there were no significant differences of mean age and sex distribution between Child-Pugh classes (Table 2, *p* = 0.920), so the described effect may be real. To note that age and gender matched HGS reference values are used to identify the presence or absence of malnutrition [15, 23].

Álvares-da-Silva et al. [24, 25] suggested that in early stages of cirrhosis, muscle strength measured by HGS should be used to evaluate for malnutrition, as muscle mass measured by MAMC may not suffer changes yet. In advanced stages, MAMC will suffer changes, but it may also be overestimated due to fluid retention, hence its lack of correlation with liver disease severity. They suggested that in advanced stages, SGA may be a better tool to evaluate nutritional status.

## 5. Conclusions

Our results show that both HGS and SGA are fair predictors of disease severity and 6 months survival in cirrhotic patients. Both methods are noninvasive and easy to use in current practice.

To our knowledge, our study is the first to demonstrate the correlation between HGS and prognosis in cirrhotic patients, irrespective of etiology.

## Figures and Tables

**Table 1:** Demographic, severity, and survival data of included patients.

	Patients
Number	100
Sex ratio	
Males/females	72/28
Age, mean ± SD, years	59.2 ± 10.8
Cirrhosis etiology	
Alcoholic/viral/mixed/others^∗^	51/37/10/2
Ascites^∗∗^	
Absent/mild/moderate/severe	25/16/32/27
Spontaneous bacterial peritonitis, cases	9
Hepatorenal syndrome, cases	6
Hemorrhage, cases	12
Encephalopathy grade	
0/1/2/3/4	59/40/1/0/0
Child-Pugh score	
A/B/C	23/46/31
MELD score, mean ± SD	13.7 ± 6
MELD-Na score, mean ± SD	15.4 ± 6.5
Survival at 6 months^∗∗∗^, patients (%)	78 of 97 followed up (80.4%)

^∗^1 patient with hemochromatosis and 1 patient with Budd-Chiari syndrome. ^∗∗^Ascites from SGA points (0—absent, 1—mild, 2—moderate, 3—severe). ^∗∗∗^3 patients were lost to follow-up.

**Table 2:** Nutritional assessment values in different Child-Pugh classes.

	Child-Pugh A	Child-Pugh B	Child-Pugh C	*p* value
Patients	23	46	31	
Age, years				
Mean ± SD	60.7 ± 9.2	58.3 ± 10.5	56.2 ± 12.3	0.527
Sex ratio				
Males/females	16/7	34/12	22/9	0.920
SGA				
A/B/C	15/8/0	17/17/12	2/22/7	**<10** ^**−3**^
BMI (kg/m^2^)				
Mean ± SD	27.9 ± 5.2	26.4 ± 5.3	27.9 ± 5.9	0.522
TSF (mm)				
Mean ± SD	12.5 ± 9.1	10.3 ± 7.1	11 ± 7.1	0.646
MAC (cm)				
Mean ± SD	28.5 ± 5.1	25.8 ± 4.5	26.5 ± 4.8	0.175
MAMC (cm)				
Mean ± SD	28.1 ± 4.8	25.4 ± 4.3	26.1 ± 4.7	0.170
HGS (kg)				
Mean ± SD	35.6 ± 12.4	29.3 ± 10.1	25.3 ± 7.8	**0.007**

**Table 3:** Comparisons between mean MELD and MELD-Na scores among SGA classes.

	MELD		MELD-Na	
	Mean ± SD	*p* value	Mean ± SD	*p* value
SGA		**0.003**		**0.013**
A	11.4 ± 5.3		13.2 ± 6	
B	14.7 ± 6.4		16.4 ± 6.6	
C	15.4 ± 5.7		17.5 ± 6.9	

**Table 4:** Correlations between MELD and MELD-Na and BMI, MAMC, and HGS.

	MELD		MELD-Na	
	Pearson *r* coefficient	*p* value	Pearson *r* coefficient	*p* value
BMI	0.047	0.648	0.03	0.976
MAMC	−0.073	0.482	−0.131	0.202
HGS	−0.255	**0.012**	−0.270	**0.008**
